# Sex influenced association of directly measured insulin sensitivity and serum transaminase levels: Why alanine aminotransferase only predicts cardiovascular risk in men?

**DOI:** 10.1186/s12933-015-0222-3

**Published:** 2015-05-20

**Authors:** Barbara Buday, Peter Ferenc Pach, Botond Literati-Nagy, Marta Vitai, Gyorgyi Kovacs, Zsuzsa Vecsei, Laszlo Koranyi, Csaba Lengyel

**Affiliations:** Drug Research Center, Department of Metabolism, Balatonfüred, Hungary; Tritoo Informatics Ltd., Balatonfűzfő, Hungary; Faculty of Medicine, First Department of Internal Medicine, University of Szeged, Szeged, Hungary

**Keywords:** Alanin aminotransferase, Insulin sensitivity, Gender difference

## Abstract

**Background:**

Non alcoholic fatty liver disease (NAFLD) is an independent cardiovascular (CV) risk factor which is closely associated with insulin resistance measured by both direct or indirect methods. Gender specific findings in the relationship between alanine aminotransferase (ALT) and CV disease, the prevalence of NAFLD and type 2 diabetes (T2DM) have been published recently.

The aim of the present study was to explore the gender aspects of the association between insulin sensitivity, liver markers and other metabolic biomarkers in order to elucidate the background behind the sex influenced difference in both NAFLD, T2DM and their association with CV risk.

**Patients and methods:**

158 female (47 normal and 111 impaired glucose intolerant) and 148 male (74 normal and 74 impaired glucose tolerant) subjects were included (mean age: 46.5 ± 8.31 vs. 41.6 ± 11.3, average Hba1c < 6.1 %, i.e. prediabetic population, drug naive at the time of the study). Subjects underwent a hyperinsulinemic normoglycemic clamp to determine muscle glucose uptake (M_3_), besides liver function tests and other fasting metabolic and anthropometric parameters were determined.

**Results:**

Significant bivariate correlations were found between clamp measured M_3_ and all three liver enzymes (ALT, aspartate aminotransferase and gamma-glutamyl transferase) in both sexes. When data were adjusted for possible metabolic confounding factors correlations ceased in the male population but stayed significant in the female group. Feature selection analysis showed that ALT is an important attribute for M_3_ in the female but not in male group (mean Z: 3.85 vs. 0.107). Multiple regression analysis confirmed that BMI (p < 0.0001) and ALT (*p* = 0.00991) significantly and independently predicted clamp measured muscle glucose uptake in women (R^2^ = 0.5259), while in men serum fasting insulin (*p* = 0.0210) and leptin levels (*p* = 0.0294) but none of the liver enzymes were confirmed as significant independent predictors of M_3_ (R^2^ = 0.4989).

**Conclusion:**

There is a gender specific association between insulin sensitivity, metabolic risk factors and liver transaminase levels. This might explain the sex difference in the predictive role of ALT elevation for CV disease. Moreover, ALT may be used as a simple diagnostic tool to identify insulin resistant subjects only in the female population according to our results.

## Background

Men are well known to have a higher risk of cardiovascular disease than women. In recent years, studies have shown that adult men also have a higher risk of type 2 diabetes (T2DM) [1] and higher prevalence rates for non-alcoholic fatty acid liver disease (NAFLD) [2], while they seem to have to gain less weight to develop T2DM than do women [3]. Moreover, elevated ALT, an indicator of the presence of NAFLD is found to be a predictor of coronary heart disease (CHD) only in men [4]. T2DM is associated with increased cardiovascular risk factors in both genders but diabetic women show a greater relative increase than diabetic men [5]. It is proposed that women have to undergo greater metabolic deterioration than men to develop type 2 diabetes and as such many insulin resistance risk factors must change to a greater extent [1]. This can be explained by the notion that adult men are more insulin resistant than women [6], since women appear to have better ability to expand safer subcutaneous fat stores; hence they can remain highly insulin sensitive despite considerable weight gain [7]. This capacity is lower in men, where ectopic fat appears to accumulate more in the intra-abdominal and perivascular cells, skeletal muscle, liver and possibly pancreas.

Rising waist circumference and rising liver enzymes, in particular alanine aminotransferase (ALT) and gamma-glutamyl transferase (GGT), especially in conjunction with parallel triglyceride (TG) changes, indicate liver fat gain [8–10]. Indeed, clinical studies have associated ALT levels with insulin resistance (IR), the metabolic syndrome (MetS) and the development of T2DM [11–13]. A number of studies have reported a positive association between serum ALT levels and IR using indirect parameters such as the Homeostatic Model Assessment (HOMA-IR) [12, 13], or direct methods, such as the hyperinsulinemic normoglycaemic clamp or minimal model analysis [11, 14]. Also, there is an independent association between the hepatic IR index and ALT levels in subjects with MetS, impaired fasting glucose, impaired glucose tolerance (IGT) and T2DM [15]. In non-obese subjects, the ALT/AST ratio has been found to be the most reliable marker of IR, while in obese subjects it predicted IR better than conventional atherogenic markers such as LDL-C/HDL-C or TG/HDL-C [16].

A gender difference in the association between liver enzymes and IR was found recently in the adolescent population [17]. Although this difference is well described in the pathogenesis of T2DM in adults, and the prevalence of NAFLD, which often occurs as an accompanying disease for T2DM, gender aspects of the connection between liver function changes and directly measured insulin sensitivity in the adult population have not been addressed before, or only secondary findings indicate the existence of such difference.

The aim of our study was to carry out a sex-specific analysis in association with clamp-measured insulin sensitivity and liver function test connections in a normal glucose-tolerant/prediabetic population. We also sought to determine the gender difference in liver function changes, based on phenotypic and genetic susceptibility to MetS and T2DM. Therefore we included a cohort of healthy and prediabetic male and female subjects, genetically predisposed or not predisposed to diabetes (i.e. having a diabetic first-degree relative in the family). Male and female subjects were analyzed separately. No such data have been published before.

## Methods

### Patients

Data were retrospectively analyzed from a scientific study approved by the Hungarian Central Ethical Committee (A12988-2/2003-1018-EKU) titled” **Diagnostic investigation for the early recognition of insulin resistance syndrome and its complications (granted by Hungarian National Research and Innovation Program: NKFB -1B/0007/2002)**. Patients’ recruitment started in 2004 and ended in 2008. After obtaining signed informed consent, 158 women and 148 men were included in the study, as approved by the ethical committee. Subjects were classified based on the results of a standard 75 grams oral glucose tolerance testing (OGTT) at screening (blood drawn in the 0, 30, 60, 90 and 120 min), according to the American Diabetes Association criteria [18]. We included 47 normal glucose-tolerant (NGT) and 111 glucose-intolerant (GI) subjects in the female group; in the male group, there were 74 NGT and 74 GI subjects. Patients and healthy volunteers were recruited from our own diabetes outpatient clinic and by referral from regional GPs. All GI patients, which included impaired fasting glucose (IFG), impaired glucose-tolerant (IGT) and T2DM patients, were drug-naive at the time of the study. Patients receiving antidiabetic medication or hormone substitution therapy, or suffering from any known liver disease, were excluded from the study. Subjects with excessive alcohol consumption were also excluded, although we have excluded those who consumed even moderate or small amounts of alcohol for specific analyses of age and metabolically adjusted subpopulations.

### OGTT, IVGTT and clamp

All subjects fasted on the day of the clamp examination. They first underwent an intravenous glucose tolerance testing (IVGTT) examination to assess insulin secretion (0.3 g/bodyweight iv. glucose injection). Following the IVGTT, a hyperinsulinaemic normoglycaemic clamp examination was carried out, as described by DeFronzo et al. [19]. During a continuous infusion of insulin (45 mU × min × m^−2^) and glucose (20 %), the steady state was set at the constant glucose infusion rate (earliest from the 120th minute of clamp), where blood sugar level stayed between 5.0 and 5.9 mM/l for at least 30 min after the beginning of steady state. Glucose and insulin levels were measured from venous blood at 3-, 5-, 10-, 20-, 30-, 40-, 50- and 60 min samples of IVGTT, before the beginning, and the 0-, 10- 20-, 30 min samples of the steady state of clamp. Insulin secretion was determined from IVGTT by the insulogenic index [Δ (insulin5’-insulin3’)/Δ (glucose5’-glucose3’)] and the AIR (acute insulin response: [(insulin5’ + insulin3’)/2 – insulin0’)], both being sensitive indicators of first-phase insulin response, and hence the real beta cell function. HIRI (hepatic insulin resistance index) was estimated from the OGTT 0 and 30 min glucose and insulin values [HIRI = (GLU-AUC) × (Ins-AUC) 0-30’] described by Muhammad et al. [20]. Glucose and insulin area under the curve (AUC) values were calculated using the trapezoidal rule, both from OGTT and IVGTT. We used lean body (LB = muscle)-adjusted glucose uptake (M_3_ value, mg/min/kg_LB_) calculated from the glucose infusion rates during clamp, to measure peripheral (muscle) insulin sensitivity. Formula for calculation of serum glucose levels from mmol/l to mg/dl for the clamp M_3_ value: mg/dl = 18 × mmol/l. Body composition was determined by dual-energy X-ray absorptiometry (DPX-MD+, GE-Lunar, USA, Florida).

### Biochemical measurements

Routine biochemical parameters were measured on Cobas Mira and Hitachi 912 laboratory automats with the same method (according to IFCC recommendations) during the recruitment period (2003 – 2008). Reference ranges, detection limits and test principles were unchanged during this test period. Alanine aminotransferase (ALT), aspartate aminotransferase (AST), gamma-glutamyltransferase (GGT), alkaline phosphatase (ALP), serum bilirubin, (ALP and serum bilirubin only used in feature selection analysis), free fatty acid (FFA), insulin, glucose, HbA1c levels and conventional lipid parameters were determined using Roche reagents (Roche Diagnostics, Germany).

Measurements of specific serum parameters (such as hormones, lipid fractions etc.) were performed at the same time and with the same tests. Total estradiol, testosterone, FSH and serum insulin levels were measured with an Elecsys 2010 electrochemiluminescense automat (Roche Diagnostic, Germany). Serum leptin, adiponectin, interleukin-6 (IL-6) and tumor necrosis factor-*α* (TNF-*α*) levels were measured by the enzyme-linked immunosorbent method (Quantikine DLP00, Quantikine DRP300, Quantikine HS600B and Quantikine HSTA00D kits respectively; R&D Systems, Minneapolis, MN, USA,). Lipid fractionation was done by the Lipoprint System® (Quantimetrix, USA). Lipid subfractions (very low density lipoproteins [VLDL], intermediate-density lipoproteins [IDL-A, −B and –C], and low-density lipoproteins [LDL1 − 4 subfractions, LDL 2–4 subfractions = small-dense LDL]), total LDL and high-density lipoprotein [HDL] were separated by gel electrophoresis.

### Statistics

All statistical analyses were performed with R Statistical Software (version 3.1.0). The calculated descriptive statistics was the mean, standard deviation, median and mean absolute deviation (MAD) for each value presented as not all variables were normally distributed. Sample size determination was done empirically based on other clamp studies in the original protocol. We have used boot strap analysis (Monte-Carlo simulation) to test the minimal sample number to determine statistical differences between groups. The Wilcoxon rank sum test was used to assess group differences of biochemical and anthropometric parameters as most variables were not normally distributed. For Spearman’s correlation coefficients were calculated to determine the strength of association between liver enzymes and other metabolic parameters. Partial correlation coefficients were used to assess the influence of age, body mass index (BMI), body fat percentage (BFP), HbA1c, FSH (women) and alcohol consumption on significant correlations. A p value of ≤0.05 was considered significant.

After screening the male and female population for specific filters, homogenous subpopulations were formed matched for general metabolic features in both sexes (i.e. subpopulations did not differ in age, BMI, body fat percent, AC and HbA1c), when comparing subjects genetically disposed and not disposed to diabetes (GD vs. GND groups). Wilcoxon rank sum test test was used to compare selected subpopulations for individual features as not all parameters were normally distributed.

The Boruta algorithm was used to find the most important attributes that are related to the M_3_ value. This algorithm is a wrapper built around the randomForest classification algorithm (implemented in the R package randomForest) [21]. The randomForest algorithm is an ensemble approach (divide and conquer approach); it grows many decision trees and it gives a numerical estimate of the importance of a feature. A Z score is used as the importance measure since it takes into account the fluctuations of the mean accuracy loss among trees in the forest. To avoid random fluctuations in determining the importance of any given attribute, a reference set of ‘shadow attributes’ is used for deciding which attributes are truly important, since the importance of a shadow attribute can be non-zero only due to random fluctuations [22].

Multiple regression analysis was carried out in order to determine the ability of the attributes selected by the Boruta algorithm to predict clamp M_3_. Multiple linear regression models as functions of explanatory variables were identified for men and women groups (see ‘Results’).

## Results

General characteristics of the population are shown in Table 1. Mean HbA1c values were under 6.1 % in all groups, i.e. the population consisted of either normal glucose tolerant or mostly prediabetic (IGT/IFG or fresh T2DM) subjects, both slightly overweight and obese individuals. Men tended to be younger and slightly more insulin sensitive than women in both (NGT and GI) groups, although there were a lower prevalence of genetic predisposition in the NGT male group. The prevalence of genetic predisposition (the presence of diabetes in 1^st^ degree relatives, GD vs. GND groups) were between 20 and 40 %, lowest in the male NGT group, as indicated. Significant differences were found between age and metabolic parameters in NGT vs. GI groups in both genders as expected. ALT and GGT levels were higher in the GI vs. NGT groups in both sexes, AST levels differed significantly only in the male group between NGT and GI subjects.

**Table 1:** Baseline biochemical and clinical characteristics of male and female populations. All values are means, medians and mean absolute deviation (MAD)

	NGT males (n = 74)			GI males (n = 74)		
	Mean ± SD	Median	MAD	Mean ± SD	Median	MAD
Age (years)	33.43 ± 11.60	30.00	10.46	48.72 ± 9.33**	51.40	7.75
BMI (kg/m2)	26.66 ± 5.01	25.10	2.42	29.99 ± 4.30**	29.41	4.01
AC (cm)	94.54 ± 13.11	90.50	6.67	105.07 ± 14.55**	105.00	9.64
HbA1C (%)	5.41 ± 0.43	5.40	0.44	5.90 ± 0.68**	5.80	0.59
Glu-0 (mmol/L)	4.89 ± 0.72	4.83	0.56	6.09 ± 1.04**	5.96	0.96
M3 (mg/min/kg)	8.83 ± 3.10	8.81	2.82	5.88 ± 2.77**	5.74	2.38
HIRI	54.42 ± 33.06	48.86	24.98	63.86 ± 33.84**	60.24	32.09
TG (mmol/l)	1.46 ± 1.14	1.07	0.55	2.65 ± 2.18**	2.00	1.33
HDL-C (mmol/L)	1.35 ± 0.40	1.36	0.36	1.13 ± 0.42**	1.08	0.29
LDL-C (mmol/L)	2.48 ± 0.83	2.31	0.80	3.00 ± 1.05**	3.03	0.79
AST (U/L)	21.68 ± 5.44	20.00	4.45	27.57 ± 12.93**	25.00	8.90
ALT (U/L)	24.81 ± 10.56	22.00	7.41	36.24 ± 26.98**	29.50	15.57
GGT (U/L)	26.93 ± 15.73	22.00	10.38	48.44 ± 33.50**	39.00	28.17
Alcohol (g/day)	0.09 ± 0.38	0.00	0.00	0.30 ± 0.67**	0.00	0.00
Hypertension (%)	10.81	NA	NA	43.24	NA	NA
Smoking (%)	14.86	NA	NA	20.22	NA	NA
Genetic predisposition (%)	21.62	NA	NA	32.89	NA	NA
	NGT females (n = 47)			GI females (n = 111)		
Age (years)	45.10 ± 10.39	46.00	10.43	50.80 ± 8.54**	53.00	7.41
BMI (kg/m2)	26.85 ± 4.25	26.57	4.74	31.49 ± 5.25**	31.57	4.96
AC (cm)	91.95 ± 12.18	92.00	14.08	104.42 ± 12.49**	103.00	11.86
HbA1C (%)	5.62 ± 0.50	5.60	0.59	6.06 ± 0.63**	6.02	0.62
Glu-0 (mmol/L)	5.08 ± 0.49	5.08	0.44	5.75 ± 0.77**	5.65	0.76
M3 (mg/min/kg)	6.64 ± 3.24	6.29	2.79	4.36 ± 2.08**	3.92	1.69
HIRI	63.07 ± 32.51	54.76	27.80	75.23 ± 49.42**	60.77	36.46
TG (mmol/l)	1.43 ± 0.83	1.24	0.43	1.79 ± 0.81**	1.57	0.74
HDL-C (mmol/L)	1.48 ± 0.55	1.49	0.61	1.33 ± 0.51	1.27	0.36
LDL-C (mmol/L)	2.65 ± 0.81	2.51	0.61	3.20 ± 1.06**	3.17	0.87
AST (U/L)	23.00 ± 9.76	21.00	5.93	23.75 ± 10.37	20.00	4.45
ALT (U/L)	21.79 ± 14.21	20.00	10.38	25.33 ± 12.98*	22.00	8.90
GGT (U/L)	25.95 ± 28.13	19.00	11.86	31.76 ± 25.29**	25.00	11.86
Alcohol (g/day)	0.14 ± 0.55	0.00	0.00	0.02 ± 0.15	0.00	0.00
Hypertension (%)	23.40	NA	NA	43.24	NA	NA
Smoking (%)	14.86	NA	NA	18.18	NA	NA
Genetic predisposition (%)	21.62	NA	NA	36.36.	NA	NA

Significant differences between groups are indicated (Wilcoxon rank sum test, *: p < 0.05, **: <0.01). The prevalence of genetic predisposition to T2DM, hypertension is indicated in percentage

**Fig. 1:**
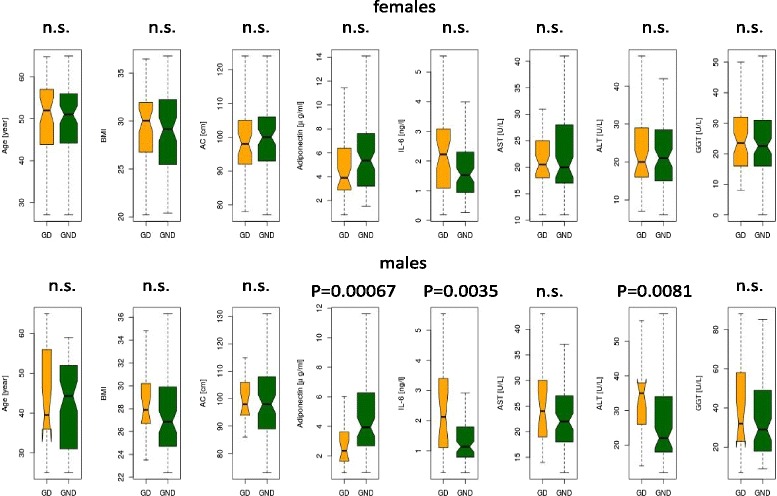
Boxplot diagrams for comparison of metabolic parameters and liver enzymes, in age and BMI matched GD and GND males and females. Homogenous subpopulations were formed for group comparisons between GD and GND groups with Wilcoxon test (GD males *n* = 26, GND males *n* = 60, GD females *n* = 58, GND males *n* = 91). No significant differences were measured between age, BMI, abdominal circumference between GD and GND groups. Moreover body fat percentage, insulin sensitivity (M3), OGTT-glucose, estradiol, testosterone and FSH levels did not differ significantly between groups (data not shown). Even small or moderate alcohol consumption was excluded. In age and BMI matched GD males ALT, adiponectin and IL-6 values were significantly higher than in GND males. Further significant differences were found in HDL-C in males. In females no significant difference was noted, except for IL-6, which was border significant (*p* = 0.063)

Following the exclusion of subjects with even mild to moderate alcohol consumption, age and BMI matched homogenous subpopulations either with or without diabetic genetic background (GD vs. GND group) were compared within sexes as described in “*Statistics”*. Age, BMI and abdominal circumference did not differ significantly between groups (Fig. 1). Furthermore, body fat percent, HbA1c, M_3_ and sex hormone levels were also similar (data not shown). In males, significant difference was found between ALT levels of GD and GND subgroups (Fig. 1). Moreover, IL-6 levels were significantly higher, adiponectin and HDL-C levels (median: 1.04 vs. 1.20 mmol/L, *p* = 0.0038) were significantly lower in GD males compared to GND males by the Wilcoxon test (all data are shown on Fig. 1, except for HLD-C). No difference was noted in the female group for any of these variables, except for IL-6 which was somewhat lower although not significantly different in GD compared to GND females (median: 2.28 vs. 1.62 ng/mL, *p* = 0.063).

**Fig. 2:**
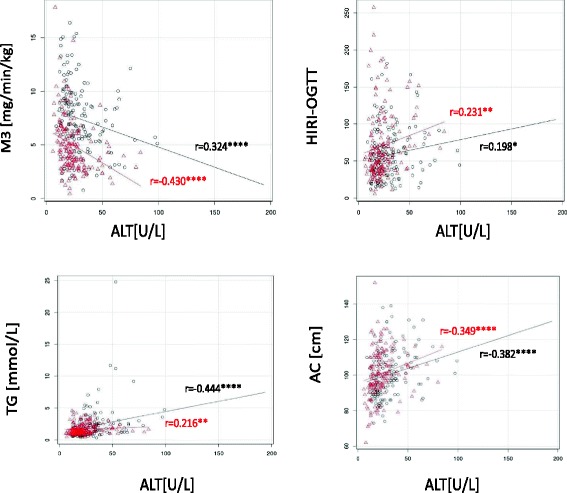
Scatter plots for bivariate correlations between liver enzymes and component of metabolic syndrome. Scatter plots in men (black spots) and women (red spots) representing bivariate (Spearman) correlations between HIRI, M3, basal glucose, TG, AC and liver enzymes (ALT, AST, GGT). Correlation coefficients are indicated in black (men), and in red (women). Correction done for BMI, age, HBA1c, genetic disposition and alcohol consumption. Significance level of each correlation is further indicated *: p < 0.05, **: p < 0.01, ***: p < 0.001, ****: p < 0.0001

Scatter plots with Spearman correlation coefficients are shown in Fig. 2 between M3, HIRI_OGTT_, TG, abdominal circumference (i.e. major components of metabolic syndrome) and ALT. Furthermore, simple bivariate and partial correlation coefficients are listed in Table 2 between liver enzymes (AST, ALT and GGT) and metabolic parameters (including M_3_, HIRI, blood sugar level, insulin secretion, lipids and adipocytokines), after correcting for age, BMI, alcohol consumption, HbA1c, abdominal circumference (and FSH in females). In males triglyceride, HDL-cholesterol, free fatty acid and AIR show significant correlations with ALT (and AST) after adjusting with the above confounding factors, while in females it is the clamp measured glucose uptake per se along with blood sugar values that stay significantly related after correction is done (see Fig. 2 and Table 2). GGT is rather non sex-specific, i.e. corrected associations with GGT show a similar pattern in both genders.

**Table 2:** Bivariate correlations adjusted for confounding factors between metabolic parameters and liver enzymes in male and female subjects

	AST (U/L)				ALT (U/L)				GGT (U/L)			
	Males		Females		Males		Females		Males		Females	
	R	Partial R	R	Partial R	R	Partial R	R	Partial R	R	Partial R	R	Partial R
HbA1c (%)	n.s.	n.s.	n.s.	n.s.	n.s.	n.s.	0.204*	n.s.	n.s.	n.s.	0.229**	n.s.
Glu-0 (mmol/L)	0.244**	n.s.	n.s.	n.s.	0.268***	n.s.	0.31****	0.177*	0.360****	0.25**	0.335****	0.214***
AIR (uU/mL)	−0.181*	n.s.	n.s.	n.s.	−0.189*	−0.201*	n.s.	n.s.	−0.181*	n.s.	n.s.	n.s.
FFA-0 (mmol/L)	0.326****	0.234*	0.206*	n.s.	0.295***	0.203*	0.276**	n.s.	0.385****	0.185*	0.277***	0.198*
M3 (mg/min/kg)	−0.167*	n.s.	−0.311****	0.216***	−0.324****	n.s.	−0.430****	−0.270***	−0.323****	n.s.	−0.337****	−0.268***
HIRI	n.s.	n.s.	n.s.	n.s.	0.198*	n.s.	0.231**	n.s.	0.193*	n.s.	0.240**	n.s.
TG (mmol/L)	0.389****	0.315***	0.201*	n.s.	0.444****	0.288**	0.216**	n.s.	0.636****	0.525****	0.299****	0.200*
HDL-C (mmol/L)	n.s.	n.s.	n.s.	n.s.	−0.255**	−0.218*	n.s.	n.s.	n.s.	n.s.	n.s.	n.s.
LDL-C (mmol/L)	n.s.	n.s.	n.s.	n.s.	0.168*	n.s.	n.s.	n.s.	0.330****	0.244**	0.167*	n.s.
Leptin (ng/mL)	n.s.	n.s.	0.239**	0.198*	0.288***	n.s.	0.166*	n.s.	0.292***	n.s.	n.s.	n.s.
Adiponectin (ug/mL)	n.s.	n.s.	n.s.	n.s.	−0.219**	n.s.	n.s.	n.s.	−0.164*	n.s.	n.s.	n.s.
IL-6 (ng/mL)	0.180*	n.s.	n.s.	n.s.	n.s.	n.s.	n.s.	n.s.	0.245**	n.s.	n.s.	n.s.

Significant Partial correlations are indicated after adjustment for age, BMI, abdominal circumference, body fat percent and alcohol consumtion. In females bivariate correlations are also corrected for FSH levels. *: <0.05, **: <0.01, ***: <0.001, ****: <0.0001

**Fig. 3:**
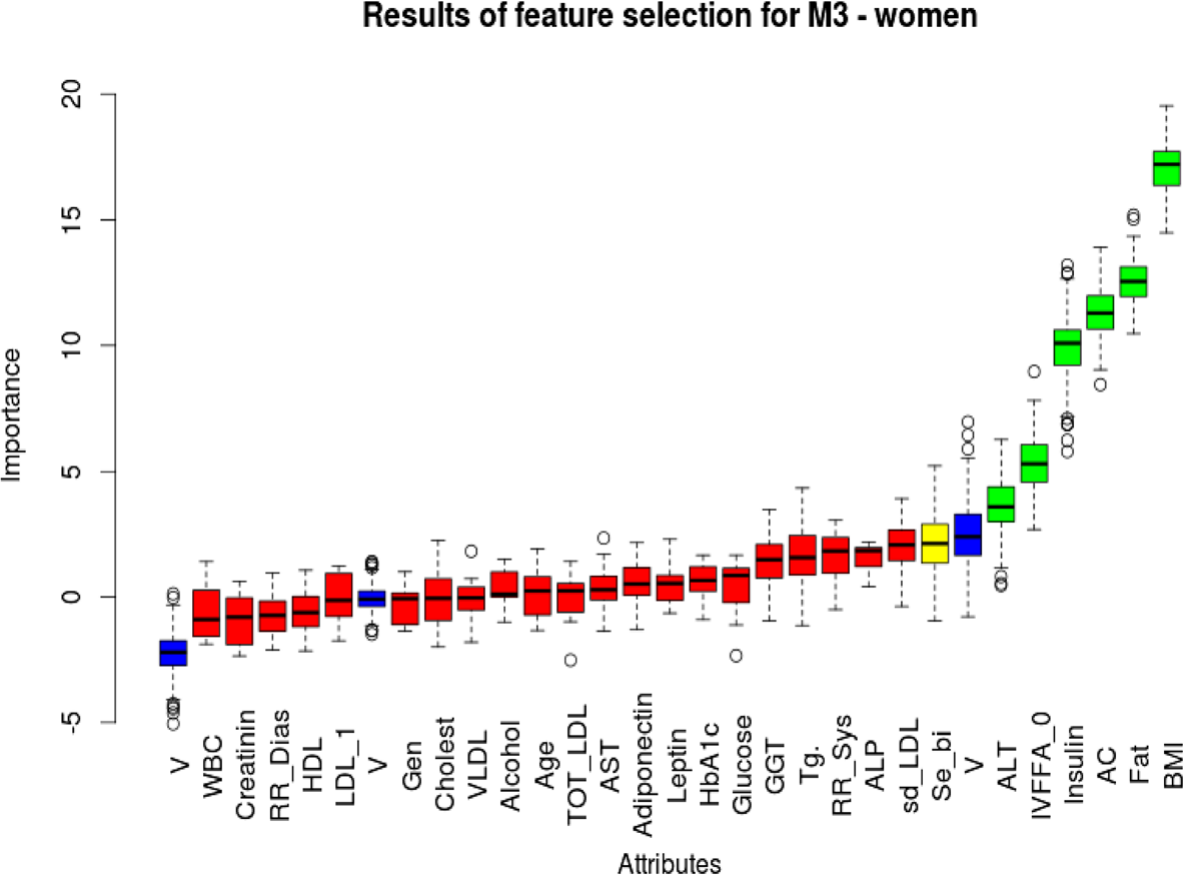
Feature selection (Boruta algorithm) analysis for M_3_ in women. Important attributes are marked in green: BMI, fat percentage, abdominal circumference, insulin, basal FFA (IVFFA_0) and ALT (mean Z: 18.46, 12.04, 9.34, 5.76 and 3.85, respectively). Yellow and red columns represent attributes that were rejected or ‘tentative’ as being important for M_3_: these are (in order of importance): serum-bilirubin, small-dense LDL, alkaline phosphatase, systolic blood pressure, triglyceride, GGT, fasting glucose, HbA1c, leptin, adipoectin, AST, total-LDL-cholesterol, age, alcohol consumption, VLDL, total-cholesterol, genetic predisposition, LDL-1 subclass, HDL subclass, diastolic blood pressure, creatinin, white blood cell count. Mean, median, minimum and maximum Z values are represented on ‘Y’ axis

**Fig. 4:**
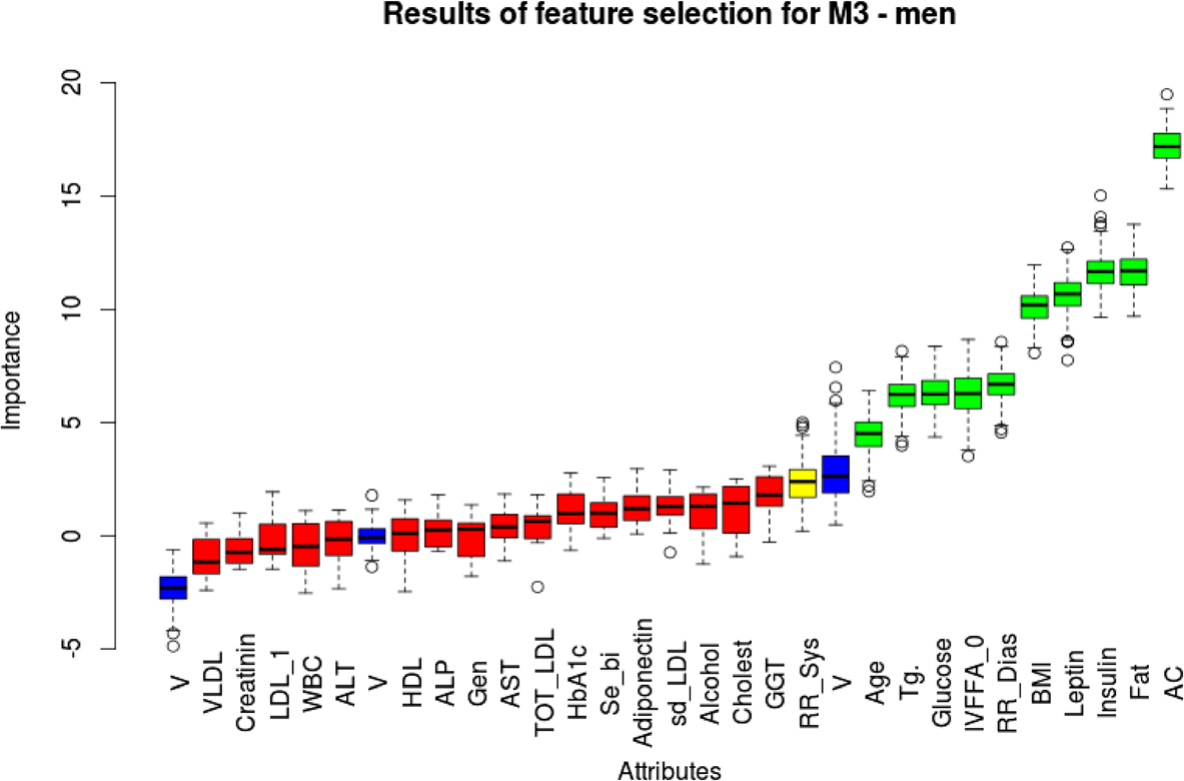
Feature selection (Boruta algorithm) analysis for M_3_ in men. Important attributes are marked in green: abdominal circumference, insulin, body fat percentage, leptin, BMI, diastolic blood pressure, TG, basal FFA, serum glucose and age (mean Z values: 16.65, 13.08, 12.19, 11.77, 6.12, 6.00, 5.39, 4.92 and 4.11, respectively). Yellow and red columns represent attributes that were rejected or ‘tentative’ as being important for M_3_. These are (in order of importance): systolic blood pressure, GGT, total cholesterol, alcohol consumption, small-dense LDL %, adiponectin, serum-bilirubin, HbA1c, total-LDL %, AST, genetic predisposition, alkaline phosphatase, HDL %, ALT, white blood cell count, LDL-1 %, creatinin, VLDL %. Blue columns represent ‘shadow attributes’. Mean, median, minimum and maximum Z values are represented on ‘Y’ axis

Feature selection analysis (Boruta algorithm) confirmed the difference between sexes (Fig. 3 and Fig. 4). The analysis was carried out separately in males and females to determine the list of ‘important attributes’ for M_3_, determined by the “Z” value (axis Y: mean, median, minimum and maximum values). ALT proved to be an ‘important’ attribute for M_3_ only in females besides BMI, BFP, AC, serum insulin and FFA levels (“Z” values see on Fig. 3). In men, on the other hand, none of the transaminase levels, instead leptin, diastolic blood pressure, TG, serum glucose and age were confirmed as ‘important variables’ besides AC, serum insulin, BFP, BMI and FFA which were common with the female group (Fig. 4).

Multiple regression analysis was conducted in order to determine the ability of the ‘important’ attributes to estimate the M_3_ values both in male and female subjects.

**Model for women**: y ~ b_0_ + b_1_x_1_ + b_2_x_2_ + … + b_5_x_5_, where response variable y is M_3_, and explanatory variables x_1_, …, x_5_ are BMI, AC, Insulin, fasting FFA, ALT, respectively, and coefficients are in Table 3. The intercept b_0_ is the expected mean value of M_3_ when all x_i_ = 0. The results in Table 3 show that F = 29.95 (p < 2.2e-16), indicating that the variables collectively have a significant effect on M_3_, **ALT** and **BMI** being significant independent predictors.

**Table 3:** Multiple regression analysis for clamp M_3_ in women

Coefficients:	Estimate	Std. error	t value	Pr (>|t|)
(Intercept)	15.81509	1.22936	12.864	< 2e-16***
BMI	−0.23375	0.05272	−4.434	1.9e-05***
AC	−0.01780	0.02081	−0.856	0.39377
Insulin	−0.04213	0.02469	−1.706	0.09028.
IVFFA_0	−1.00410	0.56730	−1.770	0.07899.
ALT	− 0.03159	0.01208	−2.616	0.00991**

Signif. codes: 0 ‘***’ 0.001 ‘**’ 0.01 ‘*’ 0.05 ‘.’ 0.1 ‘’ 1

Residual standard error: 1.88 on 158° of freedom

Multiple R-squared: 0.5259, Adjusted R-squared: 0.5084

F-statistic: 29.95 on 5 and 135 DF, p-value: < 2.2e-16

**Model for men**: y ~ b_0_ + b_1_x_1_ + b_2_x_2_ + … + b_9_x_9_, where response variable y is M_3_, and explanatory variables x_1_, …, x_9_ are AC, Leptin, BMI, Insulin, TG, FFA, Glucose, RR_Dias (diastolic blood pressure) and Age, respectively, and coefficients b_1_…b_9_ are in Table 4. The intercept b_0_ is the expected mean value of M_3_ when all x_i_ = 0. The results in Table 4 show that F = 14.71 (p < 2.36e-16), indicating that the variables collectively have a significant effect on M_3_, **serum insulin** and **leptin** being the significant independent predictors.

**Table 4:** Multiple regression analysis for clamp M_3_ in men

Coefficients:	Estimate	Std. Error	t value	Pr (>|t|)
(Intercept)	19.30144	2.35704	8.189	1.9e-13***
AC	−0.03122	0.02886	−1.082	0.2813
Leptin	−0.09451	0.04291	−2.202	0.0294*
BMI	−0.07101	0.08879	−0.800	0.4253
Insulin	−0.09684	0.04147	−2.335	0.0210*
Tg	−0.07363	0.12927	−0.570	0.5699
IVFFA_0	−0.69004	0.58016	−1.189	0.2364
Glucose	−0.37848	0.23299	−1.624	0.1067
RR_Dias	−0.01234	0.02586	−0.477	0.6340
Age	−0.03591	0.01828	−1.964	0.0516

Signif. codes: 0 ‘***’ 0.001 ‘**’ 0.01 ‘*’ 0.05 ‘.’ 0.1 ‘’ 1

Residual standard error: 2.331 on 148° of freedom

Multiple R-squared: 0.4989, Adjusted R-squared: 0.465

F-statistic: 14.71 on 9 and 133 DF, p-value: 2.368e-16

**Fig. 5:**
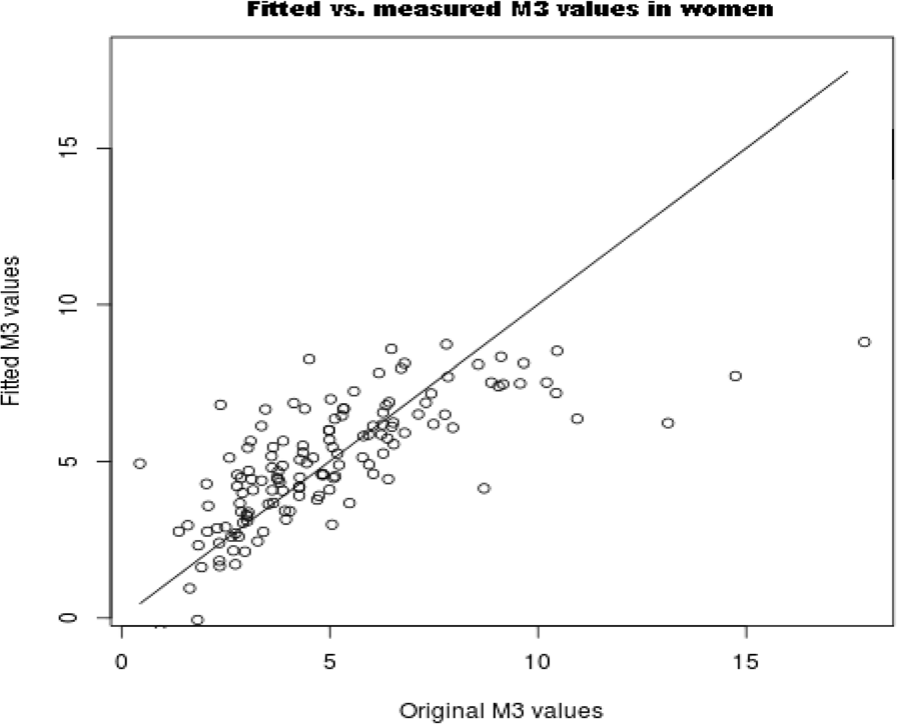
Linear regression for original vs. fitted M_3_ values in women estimated by multiple regression analysis for attributes determined by Feature Selection: BMI (*p* = 1.9e-05), AC (*p* = 0.39377), serum-insulin (*p* = 0.09028), serum-FFA (*p* = 0.07899), ALAT (*p* = 0.00991). Multiple R-squared: 0.5259, Adjusted R-squared: 0.5084

**Fig. 6:**
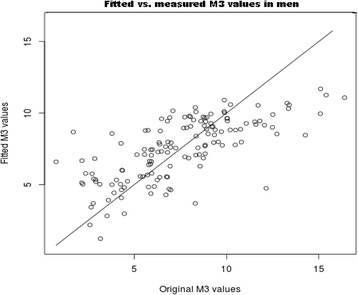
Linear regression for original vs. fitted M_3_ values in men estimated by multiple regression analysis for attributes determined by FS: AC (*p* = 0.2813), leptin (*p* = 0.0294), BMI (*p* = 0.4253), serum-insulin (*p* = 0.0210), triglyceride (*p* = 0.5699), serum-FFA (*p* = 0.2364), serum-glucose (*p* = 0.1067), diastolic RR (*p* = 0.6340), age (*p* = 0.0516). Multiple R-squared: 0.4989, Adjusted R-squared: 0.465

The ability of the ‘important’ attributes to predict measured M_3_ is indicated in Fig. 5 for women, and in Fig. 6 for men, where linear regression scatter plots for fitted vs. measured M_3_ values are shown. The regression model gave an excellent estimation of M_3_ in women, less so in men.

## Discussion

In our paper we have investigated the sex specific connection between liver function tests (ALT, AST, GGT) and insulin sensitivity measured by the gold-standard clamp method. Our main result was to demonstrate that ALT – although being in strong bivariate correlation with clamp-measured glucose uptake in both sexes – is an independent predictor of muscle glucose uptake in women but not in men.

### Gender specific aspects of NAFLD and cardiovascular risk

Many studies have previously demonstrated that ALT, AST and GGT levels independently predict type 2 diabetes and metabolic syndrome [23–27]. These markers have been shown to be associated with indirect measurements of insulin resistance, including fasting insulin levels and HOMA-IR [9, 24], and were also shown to be in conjunction with insulin sensitivity measured by the gold-standard clamp method [11]. Liver fat accumulation is closely related to liver function changes, especially ALT, therefore this enzyme is commonly used as a biomarker of NAFLD [28, 29]. Both NAFLD and T2DM have some gender aspects. Most of the studies published in this field have found that NAFLD is more common amongst men [2, 30–33]. Ayonrinde et al. have found that although the prevalence is higher in women than in men, men diagnosed with NAFLD had a more severe metabolic phenotype with higher blood glucose levels and systolic blood pressure, lower adiponectin and high-density lipoprotein cholesterol and a greater level of liver injury measures (ALT and AST levels) [34]. This supports the notion that globally deteriorating metabolic state appears to develop faster in men, independently of the presence of NAFLD. This is supported by the finding that ALT above 40U/L was an independent predictor of coronary heart disease (CHD) only in the male population in a recent study [4]. Although our results do not directly confirm this finding, the strong associations between ALT and abnormal lipid levels (TG and HDL-C) being independent from other confounding factors, which were absent in females could support these data, as atherogenic dyslipidemia is a major risk factor for CHD. Further sex-specific changes were noted in adiponectin and HDL-C levels of male subjects with diabetic relatives, both parameters being strong independent predictors of coronary disease. Even at a relatively younger age, only men having diabetic family members had significantly lower adiponectin, HDL-C and IL-6 levels than genetically not predisposed men, along with higher ALT values. This finding is consistent with the results of Feitosa et al. [4] and is not influenced by the fact that transaminase levels are usually lower in females than in males, as amongst all NGT subjects male and female data did not differ substantially (see Table 1). Although the primary aim of our study was to characterize sex specific features of the hepatic-metabolic relationship by analyzing group differences and associations rather *within* than *between* sexes, sex specific variations in transaminase levels seem to be stronger in GI than in NGT subjects (see in Table 1). This finding might be in association with that of Feitosa et al., i.e. the independent predictive value of ALT for CHD was stronger in NGT than in GI men [4].

### Gender aspects of prediabetic state, fat distribution and ALT

T2DM prevalence is higher among men than in women, especially in the middle-aged population [35, 36]. Moreover, men are diagnosed with T2DM at lower BMI than women [2]. This can be partially explained by the fact that women can remain highly insulin sensitive despite considerable weight gain as they appear to have an excellent ability to expand the safer subcutaneous fat stores [6]. In men, subcutaneous fat storage capacity is significantly lower, driven predominantly by differential sex hormone settings, thus with weight gain excess fat is placed more rapidly into other tissues in men, such as in intra-abdominal, perivascular, skeletal muscle, liver and pancreatic areas, the process being indicated by a rise in ALT and GGT along with dyslipidaemia [1].

The results of GD vs. GND group comparison (Fig. 1) suggest that progressively worsening metabolic state indicated by the presence of abnormal metabolic biomarkers characteristic of the prediabetic stage is indeed gender specific to some extent. We cannot exclude the idea that the mutual genetic background behind MetS and increased susceptibility to NAFLD [37] might be at least partially gender specific as well, although cross-sectional data would not be appropriate for drawing such a conclusion, and this notion needs further studies.

The rise of ALT and GGT, which is an indicator of liver fat accumulation, might indicate a global metabolic deterioration in men, i.e. a severe insulin-resistant state, which is further aggravated by NAFLD. The manifestation of glucose intolerance with or without insulin resistance is still compensated by the favourable hormonal environment in women, where the accumulation of intra-abdominal/visceral (liver) tissue fat is delayed by the existence of increased subcutaneous fat stores, blocking further metabolic aggravation. This theory is supported by the finding of Kang et al., who reported that the difference in ALT levels were more pronounced between normal weight obese (NWO) male and normal weight lean (NWL) male subjects than between NWO and NWL female subjects, albeit the difference was not statistically significant [38].

Similarly, the strong and independent association between FFA, TG, HDL-C and ALT in our study was only present in men and not in women, which is in accordance with the above theory. We also emphasise that female subjects were both pre- and postmenopausal at the time of the enrollment, which could at least partly explain these results, however data were corrected for age and FSH as well. Another confounding factor that could have influenced our results is the menarcheal age of the enrolled women, since earlier menarche was associated with elevated ALT, TG and CRP levels as well as increased risk of diabetes in a Brazilian study [39]. This finding needs to be further evaluated, because these data were not available in our study.

### Insulin sensitivity and liver enzymes

One of the most important findings of our study in this healthy/prediabetic population is that after the adjustment for confounding factors such as age, BMI, abdominal circumference, body fat percent, HbA1c, alcohol consumption (and FSH levels in women), all three liver enzymes (ALT, AST and GGT) stayed significantly associated with clamp-measured insulin sensitivity (i.e. muscle glucose uptake) in women but disappeared in men. This difference was only applicable for the gold standard clamp measured peripheral insulin sensitivity, i.e. the association with the estimated OGTT derived HIRI index (although stronger in females than in males) disappeared in both genders after the correction was done (see Fig. 2 and Table 2).

Analyzing data from the other aspect, a feature selection analysis-based multiple regression model has found that ALT was a significant independent predictor of clamp insulin sensitivity besides BMI in females. In men, this was fasting insulin and leptin but not liver enzyme levels.

Those studies confirming the independent association between ALT and directly measured insulin sensitivity (clamp or minimal model analysis) were carried out on either healthy, prediabetic or diabetic mixed-gender populations, although results stayed significant after the adjustment for sex and other confounding factors [11, 14]. In the study of Kawamoto et al. in a non-obese, middle-aged, mixed-gender population, the ALT/AST ratio was a better predictor of HOMA-IR than fasting insulin levels in *both* sexes [16], although in our study we did not examine the role of ALT/AST ratio. Schneider et al. did not find any sex-specific difference in the association between liver enzymes and diabetes risk. Higher ALT, AST and especially GGT predicted the incidence of diabetes in *both* genders [40]. Chen et al. found that the coexistence of obesity and ALT elevation predicted insulin resistance better than the existence of metabolic syndrome in males; however no female subjects were involved in this study [12]. Similarly, subjects with low HDL-C had higher ALT levels and increased insulin resistance (measured by the HOMA index) than subjects with high HDL-C levels, albeit no sex difference was noted [41].

On the other hand several studies have described clear gender differences in this respect. Lee et al. have described a gender difference in an adolescent population. Obesity and triglyceride were the major determinants of HOMA-IR in boys, and obesity and GGT in girls [17]. Furthermore, the independent association with IR and ALT was stronger in girls than in boys (*P* = 0.034 vs. *P* = 0.005) [17]. Poutschi et al. found a significant (p < 0.0001) linear relationship between age and ALT only in females but not in males [42] As insulin resistance increases with age, the clamp M3 – ALT independent association found only in females in our study might be contributed to this finding, even if adjustments were always done for age. In the PET CT study of Kang et al., the difference between ALT levels were more pronounced between normal weight obese and normal weight lean subjects in the male than in the female group, albeit this difference was not statistically significant [38].

A higher glutamate and glutamate/glutamine ratio, a result of abnormal ALT and AST activity was significantly associated with lower high-density lipoprotein (HDL) in women but not in men in the metabolomic study of Cheng et al. [43]. No other significant effect modification by sex was noted in the significant association between select metabolites (glutamine, glutamate and their ratio) and components of insulin-resistant phenotype.

Our results support the hypothesis that a very delicate sex difference exists in the progression/association of NAFLD with metabolic parameters in the adult population and this has an important clinical implication. In women, it is clearly evident that insulin resistance per se might indicate liver fat accumulation, and vice versa, elevated ALT levels might indicate decreased insulin sensitivity earlier than fasting insulin, lipoprotein or adipokine levels. In men, ALT (also AST and GGT) elevations coexist with other metabolic changes followed/caused by insulin resistance. Therefore liver enzyme elevation per se is not an indicator of decreased insulin sensitivity but a general metabolic deterioration along with insulin resistance in men, with no independent associations with the clamp M_3_ value.

The mechanism of these findings might be complex. The previously mentioned sex difference in fat distribution leads to increased susceptibility to intra-abdominal, visceral and liver fat accumulation in men, which is at least partially driven by differential sex hormone settings [1]. A further explanation and/or consequence is the sexual dimorphism displayed by liver-associated markers, such as sex hormone-binding globulin (SHBG) and adiponectin levels being much lower in men, consistent with their greater insulin resistance and greater risk of diabetes and cardiovascular disease at a younger age [44, 45] and the more severe metabolic phenotype at the diagnosis of NAFLD [32]. This is in agreement with the ealier mentioned finding of Feitosa et al., that ALT is a significant independent predictor of coronary heart disease in men but not in women, with the association being stronger in non-diabetic men [4].

### ALT as clamp index

There is another aspect to our findings, namely that slightly elevated ALT may strongly indicate the presence of insulin resistance in females even without hyperinsulinemia, especially in overweight women. Hence, the use of ALT in estimating clamp measured insulin sensitivity might be more relevant in females, while that of fasting insulin-based indices (i.e. the HOMA model) physiologically seems to be more appropiate in males according to our results. A gender (and racial) difference in the utility of insulin-based fasting and OGTT-based models has recently been described by Pisprasert et al., who found that gender as well as race, had a significant effect on explaining the predictability of clamp-measured glucose disposal rates (GDR) [46].

### Limitations of the study

Our study has several limitations. First, based on its cross-sectional study design, the present findings are inherently limited in the ability to eliminate causal relationships between ALT and insulin resistance or sensitivity. Second, since some of the study population had several risk factors, including hypertension, and dyslipidemia, we could not eliminate the possible confounding effect of underlying diseases on the present findings, although the prevalence of controlled hypertension, smoking and dyslipidemia were similar throughout the male and female groups. Since the original research primarily aimed the elucidation of the early diagnosis of insulin resistance rather than its association with liver disease, HVC and HBV serology was not tested at screening, so the confounding effect of latent hepatitis could not have been excluded. On the other hand the prevalence of virus carriers is low in Hungary (between 0.7-1.3 % for HCV and under 2 % for HBV) which might have had only minimal effects on the results.

## Conclusions

In our study we found that the association between liver function tests and insulin sensitivity is gender specific: muscle glucose uptake measured by the gold-standard hyperinsulinemic euglycemic clamp is independently predicted by the ALT values only in females, so ALT can be used as an indirect measure of insulin sensitivity, especially in overweight women. In males, insulin sensitivity was independently predicted only by fasting insulin and leptin levels, while the relationship with liver enzymes ceased after correction for confounding factors. Based on our results and the findings of other studies, in male patients liver fat accumulation indicated by ALT elevation is part of a general metabolic deterioration that includes, but is not limited to decreased insulin sensitivity. Therefore, ALT might be indeed an independent predictor of cardiovascular risk in men rather than in women.

Further prospective population-based studies are needed to investigate the mechanisms in order to answer these questions. This has an important clinical implication in the early diagnosis of insulin resistance and the further prevention of MetS, NAFLD and their cardiovascular complications.

## Abbreviations

AC: Abdominal circumference
AIR: Acute insulin response
ALT: Alanine aminotransferase
AST: Aspartate aminotransferase
AUC: Area under curve
BFP: Body fat percentage
BMI: Body mass index
C: Cholesterol
CHD: Coronary heart disease
FFA: Free fatty acid
GD: Genetically disposed to type 2 diabetes mellitus
GDR: Glucose disposal rate
GGT: Gamma-glutamyl transferase
GI: Glucose intolerant
GND: Genetically not disposed to type 2 diabetes mellitus
HbA1c: Glycated hemoglobin A1c
HDL: High density lipoprotein-cholesterol
HDL-C: HDL-cholesterol
HIRI: Hepatic insulin resistance index
IL-6: Interleukin-6
SHBG: Sex hormone-binding globulin
TG: Triglyceride
T2DM: Type 2 diabetes
VLDL: Very low density lipoprotein
